# A rare case of spontaneous abdominal aorta thrombosis

**DOI:** 10.1002/ccr3.4571

**Published:** 2021-08-21

**Authors:** Ahmad Samir Matarneh, Amna Sadiq, Adeel Ahmad Khan, Asma Ibrahim Eltahir, Mazen Alansari, AbdulMoqeeth Mohammed

**Affiliations:** ^1^ Department of Internal Medicine Hamad Medical Corporation Doha Qatar; ^2^ Department of Radiology Hamad Medical Corporation Doha Qatar; ^3^ Pharmacy Department‐Section of Clinical Pharmacy Hamad General Hospital Doha Qatar; ^4^ Internal medicine Department Hamad General Hospital Doha Qatar; ^5^ Internal Medicine Clinic Hamad General Hospital Doha Qatar

**Keywords:** aorta thrombosis, ischemic colitis, thrombophilias

## Abstract

Spontaneous aortic thrombosis is rare, and prompt diagnosis is needed whenever encountering a case of unexplained abdominal pain. The cause of the thrombosis needs to be evaluated thoroughly to rule out any underlying thrombophilias.

## Graphical abstract

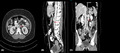

## 1 INTRODUCTION

Spontaneous abdominal aorta thrombosis is extremely rare with an unclear incidence due to its infrequent occurrence. Symptoms vary widely, ranging from being asymptomatic to a more catastrophic presentation with organ ischemia. We report the case of a 40‐year‐old lady who presented with 1‐week history of abdominal pain and was found to have spontaneous abdominal aorta thrombosis. A 40‐year‐old lady was presented to the hospital with 1‐week history of generalized abdominal pain, moderate to severe in intensity, associated with nausea and four episodes of vomiting. An initial CT scan of abdomen with contrast showed features of abdominal aorta thrombosis with ischemic colitis. Screening for an autoimmune etiology or hereditary thrombophilia was negative. The patient was treated with anticoagulation and she showed evidence of improvement in clinical symptoms and partial resolution of thrombus on follow‐up radiology. Spontaneous aortic thrombosis is rare in occurrence. Evaluation includes abdominal imaging with either CT scan or MRI, and the cause of the thrombosis needs to be evaluated thoroughly. Treatment includes anticoagulation and if needed re‐vascularization by endovascular methods.

Spontaneous abdominal aorta thrombosis is extremely rare with an unclear incidence due to its infrequent occurrence. To this date, there are very limited data on spontaneous abdominal aorta thrombosis in literature.^1^ It is of clinical significance because it can lead to devastating manifestations if it is not detected and treated in a timely manner. Symptoms vary widely, ranging from being asymptomatic to a more catastrophic presentation with organ ischemia.^2^ We present a case of a patient with severe abdominal pain who was found to have ischemic colitis secondary to spontaneous abdominal aorta thrombosis.

## 2 CASE PRESENTATION

A 40‐year‐old lady was presented to the hospital with 1‐week history of generalized abdominal pain, moderate to severe in intensity, associated with nausea and four episodes of vomiting, with no aggravating or relieving factors. There was no history of fever, hematemesis, bleeding per rectum, chest pain, or palpitations. The patient was a known case of hypothyroidism, vitiligo, and type 1 diabetes mellitus. She was a non‐smoker. The patient had no personal or family history of thrombophilia and there was no history of recurrent abortions. Physical examination revealed an afebrile patient, with a heart rate of 92 beats per minute, blood pressure 124/76 and respiratory rate 17 breaths per minute. Abdominal examination showed diffuse tenderness, no organomegaly, normal percussion note with normal bowel sounds. Cardiac, respiratory, and nervous system examination were normal.

Labs investigations showed mild neutrophilic leukocytosis, high C‐reactive proteins, microcytic anemia, normal urea, creatinine, electrolytes, and liver function tests (Table 1). Coagulation testing showed normal PT, aPTT, and INR (Table 2).

**TABLE 1:** Basic laboratory investigations

Laboratory	Reference range	Result
White blood cells	4–10*10^3^ U/L	13.2*10^3^ U/L
Hemoglobin	12–15 gm/dl	7.9 gm/dl
Platelets	150–400*10^3^ U/L	395*10^3^ U/L
Creatinine	44–80 μmol/l	33 μmol/l
Urea	2.8–8.1 μmol/l	2.1 μmol/l
Alanine transferase	0–33 U/L	6 U/L
Aspartate transferase	0–32 U/L	11 U/L
C‐Reactive protein	0–5 mg/l	145 mg/l

**TABLE 2:** Coagulation profile

Blood test	Reference range	Result
Prothrombin time	9.7–11.8 s	11.2 s
Activated partial thromboplastin time	24.6–31.2 s	24.8 s
Fibrinogen	1.7–4.2 gm/l	6.75 gm/l
Intranational normalized ratio	–	1

**FIGURE 1:**
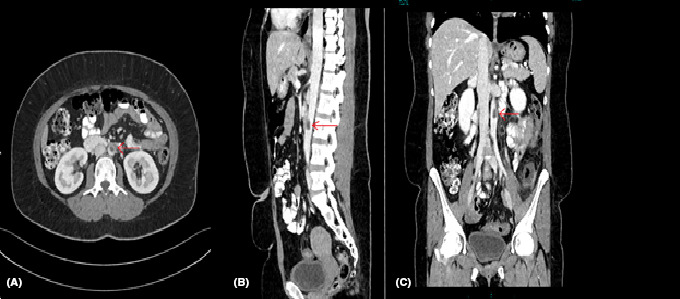
A, Axial CT. B, Sagittal CT. C, Coronal CT abdomen with contrast showing a filling defect in the abdominal aorta at the level of L3 vertebrae consistent with aortic thrombus (arrow)

**FIGURE 2:**
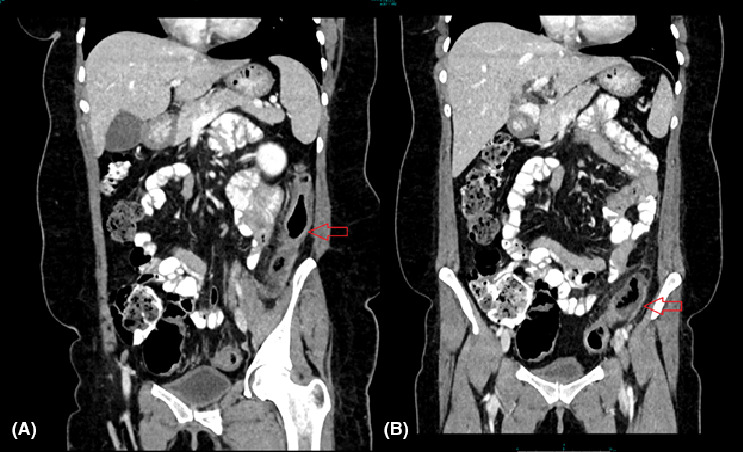
Coronal CT abdomen showing mural thickening and peri‐colonic fat stranding in A, descending colon and B, sigmoid colon suggestive of ischemic colitis

A computed tomography (CT) scan of the abdomen with contrast showed diffuse thickening of the splenic flexure, descending colon, and sigmoid colon with surrounding mesenteric edema and fat stranding as well as a thrombus in the abdominal aorta at the level of L3 vertebra (Figures 1 and 2). However, all the thrombophilia screen and autoimmune work up were negative (Table 3).

**TABLE 3:** Thrombophilia screen

Laboratory value	Reference range	Result
Lupus anticoagulant	–	Negative
Lupus screen	30.4–45.3 s	46 s
Anti‐cardiolipin antibodies		Negative
Anti‐B2 Glycoprotein antibody	–	Negative
Protein C activity	70%–140%	77.2%
Protein S activity	56.1%–126%	77.4%
Antithrombin activity	79.4%–112%	70%
Factor 2 ‐Prothrombin C	–	Negative for mutation
Factor V leiden mutation		Negative.
Anti‐nuclear antibody	–	Negative
Rheumatoid factor	0–14 IU/L	10 IU/ml
Anti DsDna Antibody	–	5.50 IU/ml negative
Antineutrophil Cytoplasmic Antibodies	–	Negative

**FIGURE 3:**
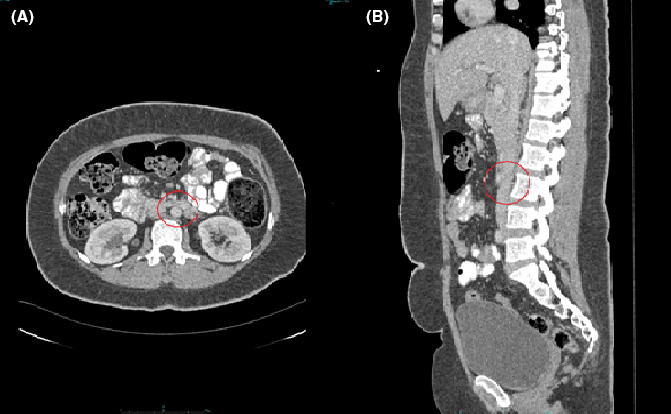
Follow‐up CT abdomen with contrast A, axial and C, sagittal view shows complete resolution of the previously seen abdominal aorta thrombus in the circled area at the level of L3 vertebrae

The patient was managed as a case of ischemic colitis due to spontaneous abdominal aorta thrombosis. She was started on anticoagulation with warfarin bridged by therapeutic dose enoxaparin with a target INR of 2–3. A follow‐up CT scan of the abdomen that was done 2 months afterward showed resolution of thrombus and improvement of the inflammatory changes in the bowel (Figure 3). Three months afterward, she presented with left‐sided abdominal pain, nausea, and vomiting. CT scan of the abdomen was done, and it showed focal segmental circumferential mural thickening of the distal descending colon as a complication of the ischemic colitis the patient was admitted and underwent left hemicolectomy. She was then discharged home after improvement.

## 3 DISCUSSION

Spontaneous arterial thrombosis is rare in occurrence, but it has a significant clinical importance as it leads to several complications secondary to blood flow impediment.^3^ It can involve any organ system with the resulting ischemia and/or infarction. Prompt diagnosis and management are always needed as any delay can cause acute complications which can include renal infarction, stroke, abdominal, and peripheral ischemia with gangrene and might eventually lead to death. Presentation depends on the system or the organ involved.^3^ Clinically significant thrombosis can be because of a primary hypercoagulable state or as secondary to trauma, immobilization, or other coagulopathic abnormalities. Suspicion should arise toward an underlying abnormality whenever a patient has history of unprovoked arterial or venous occlusion. Evaluation for causes such as factor V Leiden, antithrombin III deficiency, or protein C or S deficiency is warranted in unprovoked cases.^4^

Spontaneous abdominal aorta thrombosis is rare as the blood flowing in the aorta has a high velocity which, along with a high caliber of the artery, makes it less prone to thrombosis and it is almost always secondary to an underlying hypercoagulable status.^5^ It can present with manifestations that could range from asymptomatic in cases of chronic thrombosis to a more severe one in patients with acute thrombosis which can lead to devastating results with hemodynamic instability and even peripheral or abdominal organ loss.^6^ As the abdominal aorta is the major source of arterial supply, its involvement can lead to renal artery ischemia with renal infarction, colonic ischemia, and limb ischemia. Diagnosis should always be prompt. The initial evaluation should start with basic laboratories such as CBC, which can show increased white cell count, increased lactate denoting ischemia, acute kidney injury, or liver injury. Urgent imaging evaluation should be done next. CT scan with contrast is usually the imaging diagnostic of choice as it can reveal the thrombus and allow prompt diagnosis of any underlying complication. CT scan can also help in excluding other diagnoses.^7^ A full and thorough evaluation for possible underlying hypercoagulability must be sought. Treatment should be pursued as soon as possible, and it usually depends on the presentation and hemodynamic stability. Usually, the approach in stable patients with an established thrombus is to start on anticoagulation with warfarin and bridging enoxaparin.^8^ There are currently limited data at present on the novel anticoagulants use in hereditary thrombophilia's and in spontaneous aorta thrombosis. The duration of anticoagulation is usually guided by the underlying cause, as some patients require life‐long anticoagulation for prophylaxis.^9^

## 4 CONCLUSION

Spontaneous aortic thrombosis is rare in occurrence, and prompt diagnosis is needed whenever encountering a case of unexplained abdominal pain or embolic phenomena. Evaluation includes abdominal imaging with either CT scan or MRI, and the cause of the thrombosis needs to be evaluated thoroughly to rule out any secondary hypercoagulable states as well as evaluating for primary hypercoagulability. Treatment includes anticoagulation and if needed re‐vascularization by endovascular methods.

## CONFLICT OF INTEREST

None declared.

## AUTHOR CONTRIBUTIONS

Ahmad S Matarneh: History and physical examination, literature review, and manuscript writing. Amna Sadiq: Radiology and clinical images. Adeel Ahmad Khan: Literature review and manuscript writing. Asma Eltahir and Mazen Alansari: Clinical care. AbdulMoqeeth Mohammed: Clinical care and manuscript writing.

## ETHICAL APPROVAL

The case report was approved by Hamad medical corporation, MRC number MRC‐04‐21‐387.

## CONSENT STATEMENT

Published with written consent of the patient.

## Data Availability

The data that support the findings of this study are available from the corresponding author upon reasonable request.
